# Revisiting the Existence of EKC Hypothesis under Different Degrees of Population Aging: Empirical Analysis of Panel Data from 140 Countries

**DOI:** 10.3390/ijerph182312753

**Published:** 2021-12-03

**Authors:** Shuyu Li, Rongrong Li

**Affiliations:** School of Economics and Management, China University of Petroleum (East China), Qingdao 266580, China; s17080811@s.upc.edu.cn

**Keywords:** EKC hypothesis, aging, ecological footprint, threshold panel model

## Abstract

Population aging and environmental sustainability have become two hot topics in the world today. To clarify whether the Environmental Kuninets Curve (EKC) hypothesis between the economy and the environment is still valid in the context of population aging is the key to reveal the complexity of social-ecological systems in aging societies. So far, the impact of population aging on the environment has not been clear. To this end, an empirical analysis on the threshold panel model was conducted using panel data of 140 countries from 2000 to 2015. The global findings suggest that economic growth was the main reason for the increase in the ecological footprint at the beginning of aging. However, deepening aging weakened this association between economic and ecological footprints. For high-income countries, with the deepening of aging, the economic and ecological footprints were firstly negatively correlated, then positively correlated, and finally negatively correlated. In other words, the EKC hypothesis remained valid in high-income countries as aging deepened. In contrast, for the low- and middle-income group, the economic-environmental association was not affected by the degree of aging. This result sheds light on the variability of different income country groups in coping with the environmental impacts of aging. For the high-income group, policy makers should pay attention to the aging threshold in socio-ecological management. Only in this way can the development of aging and the ecological environment be reconciled to the greatest extent.

## 1. Introduction

In recent years, the adverse effects of climate change caused by environmental degradation have posed many threats to human survival. Similarly, the United Nations report stated that the continuous expansion of economic activities has put tremendous pressure on the ecosystem [1]. As a result, exploring the environmental changes caused by economic development plays a vital role in ecological protection. In this regard, the Environmental Kuznets Curve (EKC) was designed to illustrate the dynamic dialectical connection between economic development and environmental levels. The EKC hypothesis reveals that environmental degradation has a threshold in the path of economic development [2]. After the specific value of economic development has passed, the degree of environmental pollution begins to weaken. 

Nowadays, the world is experiencing a period of rapid increase in population aging [3]. Driven by this aging trend, is the Kuznets hypothesis between economy and environment still valid? Obviously, the change in the structure of labor supply and social consumption [4] is the main reason why the aging population affects the pattern of the global economy and ecosystem [5]. The effects of the two aspects usually superimpose together, resulting in the economic and environmental conditions in an aging society that are still unknown. As a result, studying the issue of the EKC curve under the aging trend is of positive significance for dealing with the environmental challenges brought about by the aging social crisis in the new era.

A large number of existing studies on environmental Kuznets curves typically use CO_2_ as a measure of environmental degradation [6]. However, carbon dioxide has limitations in responding to ecosystem function and ecological degradation. An increasing number of studies have chosen the ecological footprint as an indicator of the state of the environment because it is good at measuring the ecological damage and ecological carrying capacity of human activities [7]. However, studies targeting the ecological footprint have only focused on a certain geographical area and have not considered the global scale comprehensively. This gives us the feasibility of further research. To this end, this study aims to investigate the dynamic impact of global GDP on the ecological footprint in 140 countries under different stages of aging. Tests of smoothness, analysis of covariance, threshold effects tests, and threshold panel regression models are employed in this study. Further, the inclusion of control variables such as industrialization and trade level enrich the path of population aging on the Kuznets curve hypothesis. The division into different income groups also helps to reveal the effect of affluence on the existence of the EKC curve. Overall, the empirical study of this work aims to inject an aging perspective into the sustainable economic development of the new era, while providing a reference value for the next stage of ecological management. 

The structure of this study is shown below. The second section is a literature review. The Section 3 is the principle of model construction. The Section 4 is an empirical study on the effectiveness of the Kuznets curve under the background of population aging. Section 5 discusses the regression results of the global and various income groups. Section 6 is the conclusion of the full text.

## 2. Literature Review

The deterioration of the environment has made us realize that it is unsustainable to promote economic development at the expense of environmental quality. As a result, many scholars are devoted to exploring the relationship between economy and environment in order to serve coordinated development [8]. Among them, the Environmental Kuninets Curve (EKC) revealed an inverted U-shaped relationship between per capita income and environmental pollution levels, which sparked the attention of a wide range of scholars. Later, a series of in-depth studies gradually confirmed that the level of environmental pollution was weak at the early stage of economic development; with further economic development, environmental pollution showed the characteristics of first strengthening, and then weakening. However, the elasticity of the EKC curve shows different change characteristics with the different selection of environmental pollution indicators. In addition, the critical value of the economic level that makes the environmental quality from deterioration to improvement has not been conclusively determined. In the newer survey results, the ecological footprint indicator for measuring biocapacity has also begun to enter the research field of vision [9]. Thus, the literature review will sort out the research including economic and several typical environmental pressure indicators.

### 2.1. Research on the Relationship between Economic and Environmental Pressure

The research on carbon dioxide as an environmental indicator and GDP as an indicator of economic growth is abundant. Riti et al. [10] used various econometric techniques to confirm the existence of the EKC hypothesis in China, although there are some inconsistencies at the turning point. For China’s provincial level [11], the decoupling effect of the economy and carbon dioxide and its driving force vary over time [12]. Li and Jiang [13] investigated that the decoupling situation between GDP and CO_2_ in developed countries is better and more stable than in developing countries [14]. At the same time, trade openness [15] and R&D efficiency [16] effects were also the main driving forces of the decoupling process. Neves and Marques [17] analyzed the impact of the simultaneous use of traditional and alternative energy sources in the U.S. transportation sector on economic decarbonization. The calculated decoupling index emphasizes that efforts to improve energy efficiency promote environmental sustainability but are not sufficient to decarbonize the economy.

Carbon emissions are regarded as the most representative indicator of environmental pollution [18]. However, there are many indicators that have also been selected by scholars. For example, Mengual et al. [19] used the environmental footprint (EF2017) indicator to assess the sustainability of economic development. Studies believe that the environmental footprint is an indicator that reflects potential environmental impacts better than carbon emissions. Ji et al. [20] innovatively used pollutant discharge fees as an indicator to measure environmental pressure and applied it to the decoupling relationship between the economy and the environment. The decoupling relationship would also change from strong to weak. This result is confirmed in the study of the Yangtze River Economic Belt by Zhang et al. [21]. In addition, Yu et al. [22] took into account some typical air pollutants (sulfur dioxide, soot, wastewater, and solid waste emissions) indicators, and found an absolute decoupling between pollutants and economic growth. Besides, PM2.5 is often used as an indicator of environmental pressure [23].

By combing the research in this area, we found that the research on economic development and environmental pressure had a certain diversity in the choice of environmental pressure indicators. Carbon emissions were the most typical indicator. Secondly, wastewater and waste gas pollutants have also appeared in emerging research.

### 2.2. Research on the Relationship between GDP and Ecological Footprint

Recently, the ecological footprint has been widely discussed in recent years as a popular indicator for evaluating environmental development potential. Halil and Yacouba [24] analyzed data from 14 European countries from 1990 to 2014. It is found that EF as an environmental indicator is more in line with the EKC assumption than CO_2_. Similar to this conclusion, Chen et al. [25] confirmed in their research that the footprint series indicators are more suitable for identifying resource and environmental pressures. It is suggested to develop a three-dimensional ecological footprint model with footprint depth and size to discuss the spatial-temporal variation of the water carbon ecological footprint. The positive stimulus of financial development to the ecological footprint has triggered some research responses [26]. Pata [27] used cointegration and causal tests to consider the performance of globalization variables in BRICs countries’ progress. Rafael et al. [28] substituted the ecological footprint index into the research and development’s impact on environmental degradation. Sarkodie [29] used econometrics and machine learning estimation methods to confirm the existence of the scale effect hypothesis. Studies [30] have confirmed that renewable energy and trade openness have made significant contributions to overcoming environmental degradation, while economic growth bears greater responsibility for environmental damage [31]. Charfeddine [32] used the Markov exchange equilibrium model to clarify the environmental degradation under economic development from 1970 to 2015. The assessment results [33] of Brazil, Russia, India, and China show that increasing environmental sustainability requires attention to renewable energy and globalization, while being vigilant about human resources and financial development. Lanouar and Zouhair [34] re-investigated that the real per capita GDP of oil exporting countries has an inverted U-shaped relationship with EF. Yao et al. [35] innovatively incorporated corruption into the research framework of environmental carrying capacity, and affirmed the necessity of anti-corruption. In addition to the economic growth, economic globalization and financial development have also been discussed as the influencing factors of EF. Symmetrical and asymmetric ARDL methods were used [36] to reveal that the positive and negative changes of the economic globalization reduced footprint. Using G7 as a study subject, it was confirmed that economic growth and urbanization promote the ecological footprint, while financial globalization and ecological innovation reduce the ecological footprint [37]. Thus, there is a correlation between the ecological footprint, as an indicator to evaluate the spatial carrying capacity of a territory, and carbon emissions. Both are positively correlated indicators in lieu of environmental stress. The difference is that the ecological footprint is more comprehensive in terms of responding to human activities.

### 2.3. Summary of Literature

Through combing the research on environmental pressure, ecological footprint, and other indicators, the following points about the relationship between the economy and environment can be summarized. First, more and more scholars are keen to pay attention to sustainable variables under economic development. Secondly, typical empirical studies mostly use carbon emissions, waste gas, and wastewater as indicators to measure environmental degradation; however, these indicators cannot reflect the complex nature of the ecological environment. More and more studies have begun to believe that the ecological footprint is more representative of the pollution’s potential than carbon emissions. Finally, most of them have paid attention to exploring the driving factors behind environmental sustainability. Few studies take the hot spot of population aging into account between the two.

This study estimates the role of the aging process on economic and environmental sustainability from a global perspective. This provides new insights for mitigating climate change in the new era. At the same time, there are heterogeneous results between different income groups in terms of carbon emission impact factors [38], which triggers us to discuss them separately according to their respective income levels. Compared with other studies, this research has the following contributions. First, this is a new type of research that uses threshold panel estimation to discuss whether the environmental Kuznets curve hypothesis is still valid in the aging process. Second, this research adopts a multivariate empirical model and adds multiple explanatory variables to enrich the research results. Finally, this study fully considers the cross-sectional dependence among 140 countries around the world and the heterogeneity among four income groups. This systematic study made the empirical results more policy-oriented. In general, this study provides a detailed picture of the economic and environmental relationship in the context of an aging society. The research results provide new insights for exploring the applicability of the environmental Kuznets curve in the new era.

## 3. Materials and Methods

This section focuses on statistical principles and calculations for data and methods analysis. First, we analyze the theoretical basis for variable selection and introduce the tools for descriptive and covariance analysis. Second, we provide a detailed introduction to the construction of regression models and panel models.

### 3.1. Variable Selection and Data Descriptive Analysis

With the deepening of aging, will continued economic expansion lead to the excessive occupation of ecological resources? The following empirical analysis in this paper is to test the ecological footprint effects of GDP at different stages of aging. To better study the impact, this research needs to control other main factors that affect the ecological footprint. Considering the actual situation and the comprehensiveness of the data, this study selects industrial added value, the proportion of urban population, and the proportion of trade in GDP as the control variables. The reason for choosing these control variables is that in the process of economic impact on environmental pressure, the intermediate effects of industry, urbanization, and trade cannot be ignored. In addition, the social aspects reflected by these three variables are also an important source of environmental pressure.

The ecological footprint indicators of this study are selected from the Data.world database [39], and the remaining indicators are selected from the World Bank [40]. This study takes 140 countries as the research objects, and the sample time range used is 2000–2015. The specific index selection and data description are in Table 1 as follows:

(1) Explained variable: The Ecological Footprint Index is an index used to measure the geographical area required for local biological production and human activities [41], which are provided by the dataset, in units of global hectares. Since this indicator takes into account multiple dimensions such as species diversity and ecological degradation, it is regarded as a reliable indicator for assessing the sustainable development of a region [42].
(2) Explanatory variable: GDP per capita is an indicator that reflects the state of economic development from the perspective of social macroeconomic operation. This calculation did not deduct asset depreciation or natural resource depletion and degradation. The data of per capita GDP are selected from the World Bank, with constant 2010 US$ as the unit.
(3) Threshold variable: Population aging (AG) is the percentage of the population aged 65 and over to the total population, which is selected from the World Bank, and the population is determined according to the actual population definition.
(4) Control variable: Industrial value added (IND) reflects the net results obtained by social industrial enterprises after all social production activities, selected from the World Bank, with constant 2010 US$ as the unit. Urban population to total population (URB): the unit is % of total population. Trade (TR) reflects the degree to which a region’s commodities are opened to the outside world. The unit is % of GDP.

After the variables are selected, we need to perform descriptive statistics and covariance analysis on the data. Generally speaking, descriptive statistics is a generalization of the overall situation of the data. The indicators include: the sample mean ($\mathrm{mean}\;=\;\frac{x_{1}\;+\;x_{2}\;+\;\cdots \;+\;x_{n}}{n}$), standard deviation ($\mathrm{sd}\;=\;\sqrt{\frac{1}{n}\left[{\left(x_{1}\;-\;\bar{x}\right)}^{2}\;+\;{\left(x_{2}\;-\;\bar{x}\right)}^{2}\;+\;\cdots \;+\;{\left(x_{n}\;-\;\bar{x}\right)}^{2}\right]}$), most value, and median. Among them, the sample mean is used to reflect the average level of the data. The standard deviation is used to reflect the degree of dispersion of the data. The maximum and median are the maximum, minimum, and middle values of a set of data, respectively, and are obtained by ranking and are usually used to illustrate the concentration trend of a dataset.

Commonly speaking, covariance means that when the independent variables affect the dependent variable, there is a strong correlation between multiple independent variables, i.e., there is a strong substitution between the independent variables, thus leading to the problem of covariance. The analysis of covariance, on the other hand, calculates the correlation between multiple explanatory variables. The calculation formula is: $r\;=\;\frac{\sum_{i=1}^{n}\left(x_{i}-\bar{x}\right)\left(y_{i}-\bar{y}\right)}{\sqrt{{\left(x_{i}-\bar{x}\right)}^{2}}\sqrt{{\left(y_{i}-\bar{y}\right)}^{2}}}$. If the correlation coefficient value of a certain two explanatory variables is found to be greater than 0.7, one variable is removed, and then regression analysis is done. In any case, the next step of the test and regression analysis can be performed directly.

### 3.2. Construction of Threshold Regression Model

Empirical studies on the impact of the economy on the ecological footprint were mostly linear models [43]. However, since the respective stages of social development have their own characteristics, the relationship between the two may not be linear. Thus, Hansen’s threshold regression model was selected to test the nonlinear relationship between the two [44]. The data can be divided into objective intervals corresponding to the characteristics of the data according to the “self-sampling” method. This study first sets a single threshold, and further expands the setting to multiple threshold models.

In this study, the simplest panel threshold model with one value is set as Equation (1):

(1) $$\left\{\begin{array}{c} \ln EF_{i,t}\;=\;\theta_{0}\ln IND_{it}\;+\;\theta_{1}\ln URB_{it}\;+\;\theta_{2}\ln TR_{it}\;+\;\beta_{1}X_{it}I\left(q_{it}\;\leq \;\gamma \right)\;+\;\mu_{i}\;+\;\varepsilon_{i,t}\\ \ln EF_{i,t}\;=\;\theta_{0}\ln IND_{it}\;+\;\theta_{1}\ln URB_{it}\;+\;\theta_{2}\ln TR_{it}\;+\;\beta_{2}X_{it}I\left(q_{it}\;>\;\gamma \right)\;+\;\mu_{i}\;+\;\varepsilon_{i,t} \end{array}\right.$$

where the Ecological footprint represents the explained variable. $X_{it}$ Denotes the core explanatory variable GDP. Control variables include: industrial added value ($IND_{it})$, degree of urbanization ($URB_{it}$), commodity trade share ($TR_{it}$), and $\theta_{0},\;\theta_{1},\;\theta_{2}$ is the coefficient of the control variable. β is the coefficient of the main explanatory variable $X_{it}$. $q_{it}$ is a threshold variable, representing the degree of aging. I(·) is an indicative function. $\mu_{i}$ is the lag term. $\varepsilon_{i,t}$ is the error term. This formula is equivalent to Equation (2):

(2) $$\ln EF_{i,t}\;=\;\left\{\begin{array}{l} \theta_{0}\ln IND_{it}\;+\;\theta_{1}\ln URB_{it}\;+\;\theta_{2}\ln TR_{it}\;+\;\beta_{1}X_{it}\;+\;\mu_{i}\;+\;\varepsilon_{i,t},\;q_{it}\;\leq \;\gamma \;\\ \theta_{0}\ln IND_{it}\;+\;\theta_{1}\ln URB_{it}\;+\;\theta_{2}\ln TR_{it}\;+\;\beta_{2}X_{it}\;+\;\mu_{i}+\varepsilon_{i,t},q_{it}>\gamma \end{array}\right.$$

In other words, the model is equivalent to a piecewise function.

In addition to the single threshold value, two or three threshold values are also common manifestations. The dual threshold model is in Equation (3) as follows:

(3) $$\ln EF_{i,t}\;=\;\theta_{0}\ln IND_{it}\;+\;\theta_{1}\ln URB_{it}\;+\;\theta_{2}\ln TR_{it}\;+\;\beta_{1}X_{it}I\left(q_{it}\leq \gamma_{1}\right)\;+\;\beta_{2}X_{it}I\left(\gamma_{1}<q_{it}\leq \gamma_{2}\right)\;+\;\beta_{3}X_{it}I\left(q_{it}\;>\;\gamma_{2}\right)\;+\;\mu_{i}\;\;+\;\varepsilon_{i,t}$$

Accordingly, Equation (4) is extended to multiple threshold models:

(4) $$\ln EF_{i,t}\;=\;\theta_{0}\ln IND_{it}\;+\;\theta_{1}\ln URB_{it}\;+\;\theta_{2}\ln TR_{it}\;+\;\beta_{1}X_{it}I\left(q_{it}\;\leq \;\gamma_{1}\right)\;+\;\beta_{2}X_{it}I\left(\gamma_{1}\;<\;q_{it}\leq \gamma_{2}\right)\;+\;\cdots \;+\;\beta_{n}X_{it}I\left(q_{it}\;>\;\gamma_{n}\right)\;+\;\mu_{i}\;\;+\;\varepsilon_{i,t}$$

### 3.3. Threshold Regression of Panel Data

Since the threshold γ cannot be directly obtained in reality, γ must be estimated first. The threshold value estimation method in the threshold model is to randomly select an observation value in the threshold variable as the threshold value [45]. After repeating the above steps, the minimum sum of the squared residuals in the threshold variable is the condition for finally determining the threshold [46].

For panel data $\left(y_{it},x_{it},q_{it}:1\;\leq \;i\;\leq \;n,1\;\leq \;t\;\leq \;T\right)$, Hansen (1999) [47] considered the following fixed-effect threshold regression model (as shown in Equation (5)):

(5) $$\left\{\begin{array}{l} y_{it}\;=\;\mu_{i}\;+\;\beta_{1}{}^{\prime }x_{it}\;+\;\varepsilon_{i,t},\;q_{it}\;\leq \;\gamma \\ y_{it}\;=\;\mu_{i}\;+\;\beta_{2}{}^{\prime }x_{it}\;+\;\varepsilon_{i,t},q_{it}\;>\;\gamma \end{array}\right.$$

We suppose $x_{it}$ is explanatory variable; $y_{it}$ is explained variable, which is not related to $\varepsilon_{i,t}$. The model can be expressed more concisely as in Equation (6):

(6) $$y_{it}\;=\;\mu_{i}\;+\;\beta_{1}{}^{\prime }x_{it}\cdot 1\left(q_{it}\;\leq \;\gamma \right)\;+\;\beta_{2}{}^{\prime }x_{it}\cdot 1\left(q_{it}\;>\;\gamma \right)\;+\;\varepsilon_{i,t}$$

Assuming that the “*n*” (Number of samples) is large, and “T” (time) is small, the asymptotic theory of the large samples is based on “n→ + ∞”. We define $\beta \equiv \left(\begin{array}{c} \beta_{1}\\ \beta_{2} \end{array}\right)$, $x_{it}\equiv \left(\begin{array}{c} x_{it}\cdot 1\left(q_{it}\leq \gamma \right)\\ x_{it}\cdot 1\left(q_{it}>\gamma \right) \end{array}\right)$. Equation (6) is simplified to Equation (7):

(7) $$y_{it}\;=\;\mu_{i}\;+\;\beta^{\prime }x_{it}\left(\gamma \right)\;+\;\varepsilon_{i,t}$$

For the individual “*i*”, average the two sides of Equation (7) over time (as shown in Equation (8)):

(8) $$\bar{y_{it}}\;=\;\mu_{i}\;+\;\beta^{\prime }\bar{x_{it}}\left(\gamma \right)+\bar{\varepsilon_{i,t}}$$

Subtracting the two equations, the deviation form can be obtained by Equation (9):

(9) $$y_{it}\;-\;\bar{y_{it}}\;=\;\beta^{\prime }\left[x_{it}\left(\gamma \right)\;-\;\bar{x_{it}}\left(\gamma \right)\right]\;+\;\left(\varepsilon_{i,t}\;-\;\bar{\varepsilon_{i,t}}\right)$$

Mark:$\;y_{it}{}^{*}\equiv y_{it}-\bar{y_{it}}$, $x_{it}{}^{*}\left(\gamma \right)\equiv x_{it}\left(\gamma \right)-\bar{x_{it}}\left(\gamma \right)$, $\varepsilon_{it}{}^{*}\equiv \varepsilon_{i,t}-\bar{\varepsilon_{i,t}}$

Then: $y_{it}{}^{*}=\beta^{\prime }x_{it}{}^{*}\left(\gamma \right)\;+\;\varepsilon_{it}{}^{*}$

The corresponding residual vector is Equation (10):

(10) $${\hat{\varepsilon }}_{it}{}^{*}\;=\;y_{it}{}^{*}\;-\;\beta^{\prime }x_{it}{}^{*}\left(\gamma \right)$$

The residual sum of squares is Equation (11):

(11) $$S_{1}\left(\gamma \right)\;=\;{\hat{\varepsilon }}^{*}{\left(\gamma \right)}^{\prime }{\hat{\varepsilon }}^{*}\left(\gamma \right)$$

Chan (1993) [44] and Hansen (1997) [47] estimated the value of γ by minimizing $S_{1}\left(\gamma \right)$ corresponding to Equation (12), namely:

(12) $$\hat{\gamma }\;=\;\arg\;\min S_{1}\left(\gamma \right)$$

After obtaining the threshold estimate, two tests are required to determine the existence of the threshold.

First, test whether the threshold effect is significant:

(13) $$F_{1}=\frac{S_{0}\;-\;S_{1}\left(\hat{\gamma }\right)}{{\hat{\sigma }}^{2}}\;=\;\frac{S_{0}\;-\;S_{1}\left(\hat{\gamma }\right)}{S_{1}\left(\hat{\gamma }\right)/n\left(T\;-\;1\right)}$$

Among them, $S_{0}$ is the residual sum of squares and $S_{1}\left(\hat{\gamma }\right)$ is the residual sum of squares. ${\hat{\sigma }}^{2}$ is the residual variance. 

The next step is to test whether the estimate is statistically significant. This paper used the Bootstrap test to test whether empirical distribution meet a specific likelihood ratio, as in Equation (14) [48]:

(14) $$LR_{1}\left(\gamma \right)\;=\;\frac{S_{1}\left(\gamma \right)\;-\;S_{1}\left(\hat{\gamma }\right)}{{\hat{\sigma }}^{2}}$$

After the first threshold is calculated, it is necessary to continue to calculate whether the search has a double threshold according to the same steps, and then to check whether there are more thresholds, until no new threshold is tested, and the test ends.

## 4. Empirical Study

This section mainly discusses the environmental sustainability effects under the threshold of aging from the perspective of empirical analysis. Based on the collected data, this study selects 2000–2015 as the sample selection interval. According to the processing process of the panel regression model, this research first conducts statistical analysis and unit root test on the data, aiming to analyze the basic characteristics and judge whether premise of processing is satisfied. Second, a threshold panel regression model was constructed for 140 countries worldwide to identify the characteristics of economic development affecting ecological footprints under different aging stages. Finally, we further explored the heterogeneous effects of GDP on ecological footprints by different income groups separately.

### 4.1. Data Preprocessing

#### 4.1.1. Descriptive Analysis

Descriptive analysis of data mainly describes the overall situation of the quantitative data by analyzing the characteristics of the concentration and volatility of data. Therefore, research usually first conducts descriptive analysis, and then conducts in-depth analysis on this basis.

We performed logarithmic processing on individual variables to standardize the data. According to descriptive statistics (as shown in Table 2), the ecological footprint of 140 countries in the world is approximately 12.076 to 22.383, with an average value of 16.988. The proportion of the global elderly population is between 0.685 and 26.019, with an average of 7.883. The mean value of the main explanatory variable GDP is 8.393, and the standard deviation is around 1.5. In addition, among the control variables, the standard deviation of industrialization is larger than that of urbanization and commodity trade, which shows that the variable of industrialization is obviously different on a global scale. The standard deviation reflects the degree of dispersion of the data series. The larger the value, the greater the sample difference.

It is worth noting that, in terms of the standard deviation values of all variables, aging shows more obvious regional differences than other variables. Therefore, the use of aging as the threshold variable in this study has some theoretical basis and practical significance.

In addition, the problem of multicollinearity is to say that a change in one explanatory variable causes a change in another explanatory variable. When the problem of cointegration occurs among the explanatory variables, it may lead to the sign of the regression coefficients being exactly opposite to the actual situation. Therefore, panel regression models require no multicollinearity between the main explanatory variables as well as the control variables. In this study, the correlation coefficients between the variables are solved to determine the co-linearity between the explanatory variables. According to statistical principles, cointegration is considered to exist when the correlation coefficient between two variables is greater than 0.7. “LN” stands for taking the logarithm of the data. As shown in Table 3, the highest correlation coefficient between the variables was 0.657, thus meeting the conditions for establishing the regression model.

#### 4.1.2. Unit Root Test

Non-stationary series are prone to incorrect inferences during traditional estimation. Therefore, before constructing the model, the stationarity of each variable must be tested to avoid the appearance of spurious regression.

The original tests tested for the existence of a unit root on the basis of independent identical distribution of the perturbation terms. Later, the test statistic was created according to different data processing methods, from which the IPS and LLC methods were derived. Although all methods are commonly used for panel unit root tests, there are differences in the requirements for cross-sectional data. LLC requires homogeneity of all individuals in the alternative hypothesis, while IPS allows heterogeneity of some individuals in the alternative hypothesis. The Fisher-PP test is an optimization of the above method to correct the DF statistic by a nonparametric approach in order to make it a lag estimation. In this study, the Phillips–Perron Fisher method was used to perform the unit root test (as shown in Table 4).

The first column in Table 4 is the name of each variable, columns 2–4 are the unit root test results of the original data series, and columns 5–7 are that of the first-order difference series. Columns 8–10 are that of the second-order difference processing. The t-statistic is a statistic for testing a single hypothesis for a parameter in a model. The usual t-statistic is written as t = (estimate − hypothesis)/standard error, and the usual t-statistic is obtained when the hypothesis value is zero. It is worth noting that the t-statistic is used to test whether the difference is significant. In the single-sample *t*-test, the greater the absolute value of the t-statistic, the farther the sample average deviates from the target value, that is, the greater the probability that the sample average and the target value are significantly different. In addition, the Prob indicator is a criterion to determine whether a set of variables has reached smoothness through the test. If a 95% confidence interval is used as a measure, a data series reaches smoothness when the *p*-value is less than 0.05.

The threshold panel model can be operated only when all the variables reach smoothness. From the values in Table 4, four variables in the original sequence passed the test, and six variables in the first-order difference sequence passed the test. In second-order difference, all seven variables have passed the test. Therefore, all data obey the second-order single integer, which can construct the next step of threshold effect test and regression analysis.

### 4.2. Cross-Sectional Dependence Analysis of 140 Countries

#### 4.2.1. Threshold Effect Test

The essence of the threshold regression model is to divide the sample into two groups using the threshold values. The threshold regression model is used only when the estimated parameters of the two sample groups are significantly different, otherwise it indicates that there is no threshold and using a linear model is sufficient. Therefore, before conducting the threshold panel model, we need to test the significance of the threshold effect of the aging variable. This is done by using the bootstrap sampling method (Bootstrap) to simulate the asymptotic distribution of the likelihood ratio test. The large sample distribution function of the statistic itself is transformed and calculated using Bootstrap to obtain the asymptotic *p*-value for the large sample. If the large-sample distribution of the *p*-value statistic does not conform to a uniform distribution, a threshold effect exists. Next, the number of threshold values is determined on the basis of the existence of the threshold effect. Usually, the number of threshold values is assumed first, and then the “circular method” is used to enter the model estimation. The most significant parameter is the number of thresholds selected for the study.

The results of the threshold effect test are shown in Table 5. The first column in Table 5 is the name of the threshold variable. The second column is the number of threshold values, the third column is the F-value for testing the linear relationship, and the fourth column is the *p*-value for testing the significance level of the threshold. The fifth column is the number of samplings, and the sixth to eighth columns are the critical values under different confidence intervals.

**Figure 1:**
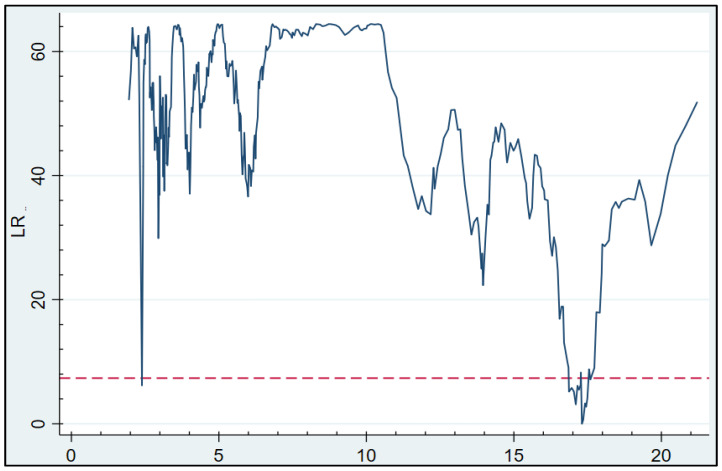
Double threshold estimate. Note: The vertical axis represents the Likelihood Ratio Sequence; the horizontal axis represents the Threshold Variable; the solid blue line represents the LR function; the red dashed line represents the 95% Critical.

From the statistical results in Table 5, it can be concluded that the *p*-values corresponding to the single, double, and triple thresholds are 0.010, 0.000, and 0.013, respectively. In the double threshold, the F-statistic is 64.417 and the *p*-value is equal to 0.000. This indicates that the double threshold has the highest level of significance in rejecting the original hypothesis. Therefore, we will perform dual threshold regression on the model, and the results of the threshold estimation regression are shown in Figure 1.

The solid blue line in Figure 1 represents the graph of the change as a function of the LR statistic, and the red dashed line represents the 95% confidence level. The intersection point between the two curves represents the threshold value at which the test is passed. As can be seen in the figure, the double threshold values for the aging variable are 17.307 and 1.871, respectively. Further, the threshold values are (2.394, 17.593) and (1.871, 1.871) at the 95% confidence interval, respectively. 

#### 4.2.2. Threshold Panel Regression Results

The previous section has passed the threshold effect test and determined the number of double threshold thresholds. Next, we perform a regression analysis of the data between the variables. For comparison purposes, this study has conducted regressions using both a fixed effects model and a threshold panel model. In the fixed effects model, CO_2_ emissions are the explanatory variable, GDP is the main explanatory variable, and industrialization, trade, and urbanization are the control variables. In the threshold panel model, the aging rate is the threshold variable, and the rest of the variables are the same as in the fixed-effects model.

The regression results are shown in Table 6. The first column is the variable name, the second column is the fixed effect model coefficient, and the third round is the threshold panel coefficient. From the regression results, the effect of GDP on ecological footprint in the fixed-effects model is positive with a coefficient of 0.0499, while in the threshold model, the effect of GDP on ecological footprint varies with the degree of aging. When aging is in the early stage of development (q ≤ 1.871), the correlation coefficient is negative −0.9597. When aging is further developed (1.871 < q < 17.593), the correlation coefficient is 0.1859. With further aging (q ≥ 17.593), the coefficient is 0.1751. For the three control variables, the correlation coefficients are significant in both types of the model’s positive values. In the fixed-effects and threshold models, the coefficients are 0.2889 and 0.2103 for industrialization, 0.0227 and 0.0294 for trade, and 0.6241 and 0.6109 for urbanization, respectively. This is consistent with the conclusion drawn by Wang et al. [49] that “urbanization does not help reduce environmental pressure.”

### 4.3. Heterogeneity Analysis of Four Income Groups

#### 4.3.1. Threshold Effect Test

Global-scale measurement has certain commonalities and cannot take into account the differences in impact caused by different income levels. Therefore, judging the impact of heterogeneity between different income groups can fully grasp the relationship between economic and ecological footprints at different stages of aging. 

This study divided 140 countries in the world into four income groups. The specific classification is shown in the Appendix A Table A1. Among them, the high-income group includes 41 countries, the middle-high-income group includes 40 countries, the middle-low-income group includes 40 countries, and the low-income group includes 19 countries.

Before panel regression, the threshold test needs to be performed. Table 7 shows results of the threshold test in different income groups. In this study, a smaller *p*-value is used as a measurement standard. Among them, the four income groups all passed the single threshold and double threshold tests, and the three income groups passed the three threshold tests.

#### 4.3.2. Threshold Panel Regression Results

In this study, the double threshold was chosen as the delineation criterion and regression analysis was conducted on the data of the four income groups (the results are shown in Table 8). As the degree of aging increased from low to high, the effect of GDP on the ecological footprint experienced a significant structural change in the high-income group, while only the coefficient size changed in the low- and middle-income group. For high-income countries, the coefficients of the impact of GDP on the ecological footprint are −1.0009, 3.368, and −0.1121 in the early, middle, and late stages of aging, respectively. For upper-middle-income countries, the coefficient of the economic impact on the ecological footprint is around 0.09 in all three stages of aging, with little variation. For lower-middle-income countries, the coefficient of economic impact on the ecological footprint decreases from a maximum of 0.4263 to 0.3457 in each of the three stages of aging. For low-income countries, instead, the economic impact on the ecological footprint experiences an increasing trend from 0.2887 to 0.3669. 

Moreover, the effects of several control variables do not differ significantly within different income groups. Specifically, the effect of industrialization on carbon emissions is more significant within the HI and UMI groups, with coefficients of 0.3999 and 0.2815, respectively, while in the LMI and LI groups, the coefficients of industrialization on carbon emissions are only 0.0658 and 0.0647, respectively. Foreign trade has a negative effect on carbon emissions within the UMI group, while in the remaining income groups, it has a positive effect. The effect of urbanization on carbon emissions is negative within the HI income group with a coefficient of −1.0608, while in the remaining income groups, the effect coefficient is positive. Overall, there are specific policy reasons behind the heterogeneous effects in countries of different income groups. This aspect will be discussed in the next section.

## 5. Results Discussion

### 5.1. Discussion of Global Panel Regression Results for 140 Countries

**Figure 2:**
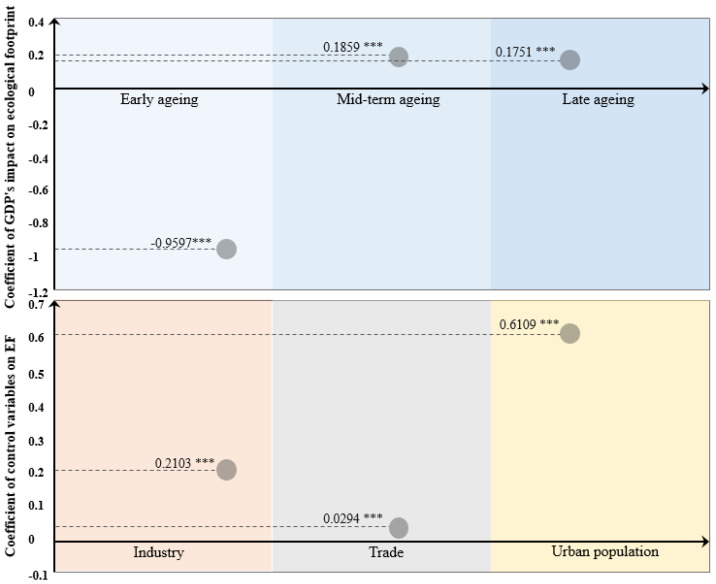
Graph of the influence coefficient of each variable in the global panel data on the ecological footprint. Note: *** represent *p*-values significant at the 1% level of significance.

Section 4.2 establishes the threshold panel data for 140 countries worldwide. Not only is the effect of economic level on ecological footprint at different aging thresholds analyzed, but also the effect of various control variables on ecological footprint is determined. The coefficients in the table are presented in Figure 2. The top side of Figure 2 shows the regression coefficients of economic development level on the ecological footprint in different social aging periods, divided into early, middle, and late aging periods, respectively. In terms of regression validity, the *p*-values of the parameter estimation tests for LN_GDP in each interval pass the 1% level of significance. In terms of regression coefficients, when aging is less than 1.871, the relationship between the economic development and the ecological footprint shows negative characteristics. That is, when aging is in its infancy, economic growth will not cause excessive ecological occupation. When aging is greater than 1.871 and less than 17.593, the correlation between the two shows positive characteristics. When aging develops to a certain degree, economic expansion causes excessive ecological occupation. Finally, when the aging is greater than 17.593, the coefficient is still a positive value of 0.1751. This means that when aging develops to a higher level, the relationship between economic development and ecological occupation has not decoupled, but the positive correlation is weakening. 

The reasons behind this phenomenon are related to the development goals at different stages. In the early 2000s, the level of aging was low, and the global economic growth rate was slow. At this time, the economic growth mode was not based on resource consumption. As aging deepens, the economic growth rate has begun to accelerate. At this time, the development model of sacrificing the ecological environment and natural resources in exchange for rapid economic growth began to emerge, resulting in a significant positive association between GDP and EF. As aging has entered an increasingly obvious stage, the deepening of aging makes this positive association weaken further. This means that the situation of resource consumption driving economic growth has begun to ease. From this perspective, the deepening of aging has led to a shift in the driving force behind economic development. In general, moderate aging can promote the coupling between economic development and the ecological footprint, while high aging can weaken the interdependence between the economic and ecological footprint.

In addition to this phenotype, the complex and close relationship between aging and social ecology provides a supporting perspective for this regression result. From a socio-ecological perspective, population aging is an important indicator of the level, quality, and prospects of social development. Thus, aging is not only the result of the joint action of multiple elements in society, but also inevitably brings about social, economic, and ecological changes in many aspects. In this study, we mainly examined the impact of economic development on the carrying capacity of the ecosystem in the context of aging. The results of the study confirm the time-series changes of the economic system on the overall carrying capacity of the ecosystem. When aging is very weak, society is in a primitive state of development (vast ecological territory and rapidly rising economy). At this time, the expanding economy does not cause a crisis in the carrying capacity of the ecosystem. Thus, economic development has a negative impact on the ecological footprint. With the increasing aging, the development state of the society changes to maturity. At this time, the expansion of population size will lead to a continued increase in the level of GDP. In this case, the rising economy will inevitably cause more ecological occupation and destruction, further increasing the ecological footprint level. At the later stages of aging, the rate of the population increase begins to slow down, and the relationship between socio-economic and ecological aspects becomes more harmonious. At this time, although economic development still brings about an increase in the ecological footprint, the magnitude of the damage becomes smaller than in the previous stage. Overall, this situation enlightens us to choose a well-structured economic development approach in response to aging. This will enable the protection of ecosystems.

### 5.2. Discussion of Panel Regression Results by Different Income Groups

**Figure 3:**
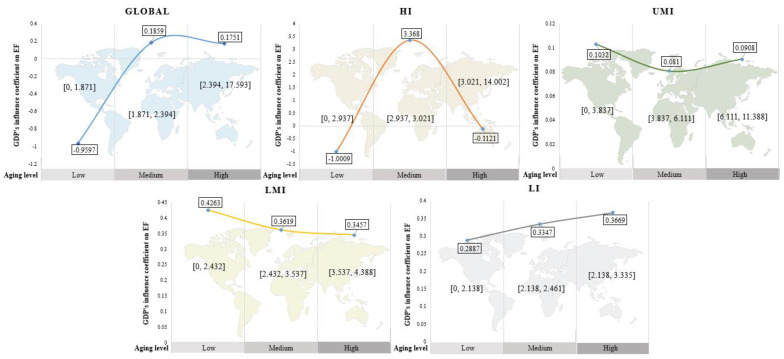
Changes in the coefficients of the impact of GDP on EF at various levels of aging in the world and in different income groups.

The dynamic relationship between economic development and social ecology at the global level has been confirmed. Further, are there differences in this phenomenon between income groups? This study divides 140 countries into four different income groups: high-income (HI), upper-middle-income (UMI), lower-middle-income (LMI), and low-income (LI). Four regression analyses were conducted in Section 4.3, which also yield the coefficients of the impact of economic development on the ecological footprint in the different groups. Figure 3 illustrates this relationship with a more detailed graph of dynamic change, where the horizontal coordinates represent the stages of development of aging, deepening from left to right. The vertical coordinate represents the impact coefficient. The curves in the figure reflect the relationship between economic development and economic development as aging changes.

The figure shows that when aging is at the initial stage of development, the negative correlation between the two is significant in high-income countries, but positive in other countries. Then, as the level of aging slowly deepens, the effect of the economy on EF is positive in all income groups. Finally, as aging deepens further, this correlation within the HI group has begun to decouple. For middle-income countries, the positive correlation tends to weaken as aging continues to deepen. In other words, in middle-income countries, economic development begins to become more ecologically beneficial in the post-aging period. For low-income countries, this positive association tends to increase as aging deepens.

The EKC curve can be derived in this study. The implication is that as aging deepens, the damage of the economic development on the ecological carrying capacity will intensify initially, and after a certain inflection point, the negative impact of this economy on the ecosystems will improve. This is further evidence that the Kuznets curve between the economy and the environment is easily established in more economically developed regions. In contrast, this theory does not apply in the middle-income and low-income groups. This view is consistent with the study of Leal [50]. However, there are also studies [51] that include the upper-middle-income groups into the scope of the establishment of the EKC hypothesis, which is different from this study due to the sample size selection. This research finding enlightens us to think about the relationship between socioeconomic and ecological balance and affluence. Generally speaking, affluent regions are more prone to social aging. In this case, a green shift in economic approach will also have a positive impact on the carrying capacity of the ecosystem.

## 6. Conclusions

Aging is a microcosm of past developmental outcomes for society and a major proposition for the world of tomorrow. This work explores the impact of increased aging on ecological sustainability. Using an aging threshold format, threshold panel regression models were developed for 140 countries globally and for four income groups. The results show the changing curves of the economic impact on the ecological footprint at different stages of aging. Several interesting findings are drawn. 

The ecological footprint (EF) reflects the extent to which human activities take over the ecosystem. The smaller the value of the “ecological footprint”, the less damage is done to nature. The global panel regression results show that an increase in GDP in the early stages of aging leads to a decrease in EF. As aging progresses, GDP growth leads to an increase in the ecological footprint, but after a certain point of inflection, this increase begins to be controlled. The panel regression results by income group show that the EKC curve between the economy and environment holds in the high-income group. For the middle-income group, the curve exhibits the second half of the inverted U-shape; for the low-income group, the curve exhibits the first half of the inverted U-shape.

This phenomenon reveals the ecological complexity behind social development. First, initial aging implies an early stage of society in which economic development is in its infancy and there is ample territorial space to absorb waste. At this time, economic growth does not bring excessive ecological occupation, and thus, economic growth has a negative impact on the ecological footprint. Second, further aging implies a massive expansion of the population. Each step of economic growth at this point leads to ecological encroachment. However, the results of the study show that this phenomenon does not last forever. The ecological appropriation by the economy will reach a critical point with the change in the structure of social development. Third, the emergence of critical points is closely related to the level of economic development through regression statistics for countries in sub income groups. The richer the country, the earlier the period of critical point emergence, and the greater the chance. Considering the importance of resource-based economic transformation in rich countries, it is reasonable to assume that the construction of a green and low-carbon society will lead to a decoupling between the economy and the environment. This reveals that it is crucial to enhance affluence and develop a green economy if we want to achieve the conservation of ecological functions along with economic development.

## Figures and Tables

**Table 1:** Variable definition and data source.

Variable Type	Variable Name	Abbreviation	Data Sources
Explained variable	Ecological footprint	EF	Data.world
Explanatory variable	GDP per capita	GDP	World Bank
Threshold variable	Aging degree	AG	World Bank
Control variable	Industry value added	IND	World Bank
	Urban population	URB	World Bank
	Merchandise trade	MT	World Bank

**Table 2:** Descriptive analysis of variables.

Variables	Mean	Sd	Min	Median	Max
LN_EF	16.9886	1.7928	12.0760	16.9489	22.3834
AG	7.8836	5.3988	0.6856	5.6422	26.0193
LN_GDP	8.3932	1.4879	5.2723	8.3318	11.6260
LN_IND	23.1171	2.4106	16.6347	22.9901	29.0568
LN_TR	4.0313	0.5163	2.0549	4.0160	5.8391
LN_URB	3.9067	0.4934	2.1097	4.0388	4.6052

Note: “Mean” stands for arithmetic mean; “Sd” stands for standard deviation; “Min” stands for minimum; “Median” stands for median; “Max” stands for maximum. “LN” stands for taking the logarithm of the data.

**Table 3:** Multi-collinear correlation coefficient of panel data.

Correlation	LN_GDP	LN_IND	LN_TR	LN_URB
LN_GDP	/	0.624787	0.272504	0.657178
LN_IND	0.624787	/	0.000737	0.580836
LN_TR	0.272504	0.000737	/	0.223213
LN_URB	0.657178	0.580836	0.223213	/

Note: “LN” stands for taking the logarithm of the data.

**Table 4:** Unit root test of panel data.

Variable	At Level			At 1st Difference			At 2nd Difference		
	t-Statistic	Prob.	Stability	t-Statistic	Prob.	Stability	t-Statistic	Prob.	Stability
LN_EF	471.754	0.000	YES	1544.46	0.000	YES	2387.88	0.000	YES
AG	134.939	1.000	NO	209.403	0.999	NO	746.274	0.000	YES
LN_GDP	275.842	0.559	YES	803.140	0.000	YES	1982.65	0.000	YES
LN_IND	275.807	0.559	NO	1034.93	0.000	YES	2180.68	0.000	YES
LN_TR	373.144	0.000	YES	1411.88	0.000	YES	2467.75	0.000	YES
LN_URB	590.928	0.000	YES	875.738	0.000	YES	1101.56	0.000	YES

Note: “t-Statistic” means statistics of the t-test; “Prob.” means concomitant probability. “LN” stands for taking the logarithm of the data.

**Table 5:** Test results of the threshold effect.

Threshold Variables	Threshold Number	F-Value	*p*-Value	Bootstrap Number	Critical Value		
					1%	5%	10%
AG	Single	76.061 ***	0.010	500	75.443	41.485	19.339
	Double	64.417 ***	0.000	500	40.302	24.598	15.402
	Triple	60.191 **	0.013	300	67.590	33.978	19.603

Note: *** represent *p*-values significant at the 1% level of significance; ** represent *p*-values significant at the 5% level of significance.

**Table 6:** Regression results of threshold model and fixed effect model.

Variables	Fixed Effect Model	Threshold Model
		AG
LN_GDP	0.0499 (0.106)	−0.9597 *** (q ≤ 1.871) (0.000)
		0.1859 *** (1.871 < q < 17.593) (0.000)
		0.1751 *** (q ≥ 17.593) (0.000)
LN_IND	0.2889 *** (0.000)	0.2103 *** (0.000)
LN_TR	0.0227(0.101)	0.0294 *** (0.030)
LN_URB	0.6241 *** (0.000)	0.6109 *** (0.000)
Constant	7.3594 *** (0.000)	8.1572 *** (0.000)
R-squared within	0.4566	0.4891
R-squared between	0.6296	0.2082
R-squared overall	0.6276	0.2097
F-test	287.86	304.04
Prob > F	0.0000	0.0000
Number of observations	2240	2240
Number of groups	140	140

Note: *** represent *p*-values significant at the 1% level of significance; “LN” stands for taking the logarithm of the data.

**Table 7:** Test results of the threshold effect.

Group	Threshold Variables	Threshold Number	F-Value	*p*-Value	Bootstrap Number	Critical Value		
						1%	5%	10%
HI	AG	Single	38.730 ***	0.006	500	34.464	19.816	13.886
		Double	84.204 ***	0.000	500	28.882	6.237	−6.719
		Triple	40.707 ***	0.000	300	29.892	19.560	14.136
UMI	AG	Single	17.587	0.118	500	49.714	29.062	19.470
		Double	17.621 *	0.082	500	64.367	25.424	14.482
		Triple	12.950	0.163	300	34.740	25.259	17.751
LMI	AG	Single	115.386 ***	0.000	500	100.894	35.747	25.009
		Double	37.176 **	0.018	500	41.387	25.704	18.641
		Triple	23.432 *	0.070	300	45.436	31.898	20.665
LI	AG	Single	44.610 ***	0.000	500	38.670	21.882	15.278
		Double	44.828 ***	0.010	500	45.100	25.714	18.335
		Triple	15.142 *	0.097	300	33.401	21.971	15.105

Note: *p*-value indicates the probability value at the corresponding F-value to determine the significance of the difference between groups; F-value indicates the statistical value of the chi-square test. *** represent p-values significant at the 1% level of significance; ** represent *p*-values significant at the 5% level of significance; and * represents p-values significant at the 10% level of significance.

**Table 8:** Regression results of the economy on ecological footprint of the threshold model in different income groups.

Variable	Regression Coefficients and Significance Levels			
	HI	UMI	LMI	LI
LN_GDP	−1.0009 *** (q ≤ 2.937) (0.000)	0.1032 (q ≤ 3.837) (0.205)	0.4263 *** (q ≤ 2.432) (0.000)	0.2887 *** (q ≤ 2.138) (0.000)
	3.3680 *** (2.937 < q < 3.021) (0.000)	0.0810 (3.837 < q < 6.111) (0.319)	0.3619 *** (2.432 < q < 4.388) (0.000)	0.3347 *** (2.138 < q < 2.461) (0.000)
	−0.1121 * (q ≥ 3.021) (0.099)	0.0908 (q ≥ 6.111) (0.263)	0.3457 *** (q ≥ 4.388) (0.000)	0.3669 *** (q ≥ 2.461) (0.000)
LN_IND	0.3999 *** (0.000)	0.2815 *** (0.000)	0.0658 ** (0.028)	0.0647 *** (0.098)
LN_TR	0.0152 (0.584)	−0.0900 *** (0.002)	0.1480 *** (0.000)	0.0833 *** (0.000)
LN_URB	−1.0608 *** (0.000)	0.5114 *** (0.000)	0.7806 *** (0.000)	1.1213 *** (0.000)
Constant	12.7952 *** (0.000)	7.9065 *** (0.000)	9.1302 *** (0.000)	8.5953 *** (0.000)

Note: *** represent *p*-values significant at the 1% level of significance; ** represent *p*-values significant at the 5% level of significance; and * represents *p*-values significant at the 10% level of significance. “LN” stands for taking the logarithm of the data.

## Abbreviations

EKC	Environmental Kuznets Curve
R&D	Research and Development
LLC	Levin-Lin-Chu test
IPS	Im-Pesaran-Shin test
ARDL	Autoregressive distributed lagged model
CO_2_	Carbon emissions
HI	High income group
UMI	Upper middle income group
BRICs	Brazil, Russia, India, China
EF	Ecological footprint
GDP	Gross Domestic Product
AG	Population Aging degree
IND	Industry value added
URB	Urban population
MT	Merchandise trade
LMI	Lower middle income
LI	Low income
G7	Group of Seven

## Appendix A

**Table A1:** Countries included in each income group (according to the classification standard provided by the World Bank).

Income Group Category	Countries
High income	ARE; AUS; AUT; BEL; CHE; CHL; CYP; CZE; DEU; DNK; ESP; EST; FIN; FRA; GBR; GRC; HRV; HUN; IRL; ISR; ITA; JPN; LTU; LUX; LVA; MUS; NLD; NOR; NZL; PAN; POL; PRT; ROU; SAU; SGP; SVK; SVN; SWE; TTO; URY; USA
Upper-middle income	ALB; ARG; AZE; BGR; BLR; BRA; BWA; CHN; COL; CRI; CUB; DOM; ECU; FJI; GAB; GEO; GRD; GTM; GUY; IDN; IRN; IRQ; JAM; JOR; KAZ; LBN; LCA; MEX; MKD; MYS; PER; PRY; RUS; SUR; THA; TON; TUR; VEN; WSM; ZAF
Lower-middle income	AGO; BEN; BGD; BOL; BTN; CMR; COG; COM; CPV; DZA; EGY; HND; IND; KEN; KGZ; KHM; LAO; LKA; LSO; MAR; MDA; MMR; MNG; MRT; NGA; NIC; NPL; PAK; PHL; SEN; SLV; STP; SWZ; TLS; TUN; TZA; UKR; UZB; ZMB; ZWE
Low income	BDI; BFA; COD; ETH; GIN; GMB; GNB; HTI; MDG; MLI; MOZ; MWI; NER; RWA; SLE; TGO; TJK; UGA; YEM
